# Neural activity in afferent projections to the infralimbic cortex is associated with individual differences in the recall of fear extinction

**DOI:** 10.1038/s41598-022-17895-5

**Published:** 2022-08-11

**Authors:** Amanda S. Russo, Ryan G. Parsons

**Affiliations:** Department of Psychology, Stony Brook University, 100 Nicolls Rd., Stony Brook, NY 11794 USA

**Keywords:** Cortex, Extinction, Fear conditioning, Neuroscience, Learning and memory, Neural circuits

## Abstract

Post-traumatic stress disorder (PTSD) is characterized by an impaired ability to extinguish fear responses to trauma-associated cues. Studies in humans and non-human animals point to differences in the engagement of certain frontal cortical regions as key mediators determining whether or not fear extinction is successful, however the neural circuit interactions that dictate the differential involvement of these regions are not well understood. To better understand how individual differences in extinction recall are reflected in differences in neural circuit activity, we labeled projections to the infralimbic cortex (IL) in rats using a retrograde tracer and compared neural activity within, and outside, of IL-projecting neurons. We analyzed these data in groups separated on the basis of how well rats retained extinction memory. We found that within IL-projecting cells, neurons in the posterior paraventricular thalamus showed heightened activity in rats that showed good extinction recall. Outside of the IL-projecting cells, increased Fos activity was observed in good extinction rats in select regions of the claustrum and ventral hippocampus. Our results indicate that differences in extinction recall are associated with a specific pattern of neural activity both within and outside of projections to the IL.

## Introduction

Fear conditioning occurs when a neutral stimulus becomes associated with an aversive, unconditioned stimulus (UCS) such that the originally neutral stimulus, now the conditioned stimulus (CS), elicits a conditioned fear response (CR) in the absence of the UCS. Extinction of conditioned fear is the reduction in the CR to the CS as a result of repeated presentations of the CS without the UCS^1^. Prior work suggests that post-traumatic stress disorder (PTSD) involves the inability to recall the extinction of a conditioned fear response^2^. The cornerstone cognitive-behavioral therapy for the treatment of PTSD is exposure therapy, which relies on the extinction of learned fear responses^3,4^. Thus, studies of individual differences in the extinction of fear rodents, and their underlying neural mechanisms, might help shed light on differences in response to trauma in humans and the treatment of PTSD. Although progress has been made in identifying the neural mechanisms that distinguish successful extinction recall from extinction failure^5,6^, there is still much to be discovered.

Rodent models are useful in this endeavor because there is marked individual variation in extinction recall among rodents^7–10^. Prior work investigating the neural mechanisms of fear extinction at the group level has suggested that activation of the infralimbic cortex (IL) is necessary for extinction recall (Refs.^11–13^, but see^14^), and a handful of studies have found reduced activity in the IL among rodents displaying poor extinction recall compared to rodents that extinguish fear well^9,15–17^. However, the mechanisms underlying the differential engagement of the IL in rodents that extinguish fear readily versus those that show poor extinction are not well understood.

One possibility is that differences in fear extinction recall among individuals is the result of differential activation of specific afferents to the IL. Anatomical studies^18^ have demonstrated that a variety of cortical and subcortical brain areas send dense projections to the IL, and in turn the IL sends efferent projections to a number of brain areas^19^. Group-level studies have found that IL projections to the amygdala are important for the acquisition of fear extinction^20–22^, whereas inputs to the IL from basolateral amygdala (BLA) have been similarly implicated in extinction learning^23^. There are fewer studies pertaining to the involvement of IL-centered circuits in extinction recall, although recent work has implicated both the ventral and dorsal hippocampus^13,24,25^ projections to the IL as being involved. Efferent projections of the IL to the thalamic nucleus reuniens also appear to be involved in the recall of fear extinction^26^.

These prior studies are beginning to paint a picture of the neural circuit interactions involved in extinction recall, however there are very little data pertaining to whether or not activity in IL-centered neural circuits drive individual differences in extinction recall. Here, we sought to determine if differences in fear extinction recall across individuals are associated with changes in the activation of input to the IL from specific brain areas. Specifically, we assessed activation of IL-afferents in the paraventricular nucleus of the thalamus (PVT), the claustrum (CLA), the BLA, and the ventral hippocampus (vHPC). These brain areas were chosen both because they send dense projections to the IL, and because there was reason to suspect they may be involved in the expression of fear extinction^18^. For example, a recent study showed that the PVT, an area known to be involved in fear acquisition and recall, is necessary for extinction recall^27^. Furthermore, previous studies have shown an increase in activity in the basal amygdala and vHPC of rats expressing extinction recall^23,28^. Finally, analysis of the claustrum was more exploratory, given that no prior work has assessed its role in extinction. However, recent work does suggest that it plays a role in contextual fear conditioning^29^.

A viral GFP-conjugated retrograde tracer was infused into the IL of the rats prior to behavioral testing, and Fos activity in IL afferents after extinction recall, fear recall, and in rats given no behavioral testing was measured. Our results indicate that projections from the posterior portion of the paraventricular thalamus to the IL show heightened activity in rats which successfully recall extinction. Outside of IL projections, neural activity within specific regions of the claustrum and ventral hippocampus was increased in rats showing good extinction. Our results indicate that patterns of neural activity, both within and outside, of projections to the IL are associated with individual differences in fear extinction recall.

## Materials and methods

### Subjects

Fifty-four adult, male, Sprague–Dawley rats (300–325 g upon arrival) obtained from Charles River Laboratories (Raleigh, NC) served as subjects. The rats were housed in pairs, with food and water freely available, on a 12 h light/dark cycle (lights on at 7 am). Two cohorts of rats were used for these experiments (n = 28 and n = 26). After exclusions for death, surgical misses, failure to express GFP in target sites, poor tissue quality, and behavioral issues (explained throughout the methods), the extinction recall group contained 21 rats, the fear recall group contained 7 rats, and the home cage control group contained 7 rats (35 total rats included in final analyses). All procedures were approved by the Stony Brook University Institutional Animal Care and Use Committee and were in accordance with the ARRIVE guidelines (https://arriveguidelines.org), and the National Institutes of Health guidelines for the care and use of laboratory animals.

### Surgery

Rats were handled for two days prior to surgery. Rats were anaesthetized with ketamine (87 mg/kg) and xylazine (10 mg/kg), and they were placed in a stereotaxic apparatus (Stoelting, Woodale, IL) and received a unilateral injection of AAVrg-CAG-GFP (Addgene,^30^) into the IL (left- and right-side injections were counterbalanced). In order to perform the injection, a 22-gauge cannula was lowered into place (AP: + 3.00, ML: ± 0.6, DV: − 5.2). A 28-gauge internal cannula (connected to an infusion pump via PE 20 tubing) was inserted into the guide cannula and used to deliver 0.6 μL of the virus at a rate of 0.15 μL per minute and stayed in place for five minutes after the infusion was complete. After suturing rats were injected with Meloxicam (1 mg/kg) and they were returned to their home cages once ambulatory. Rats stayed in their home cage for about 7 weeks to allow for recovery and retrograde transport of the virus. Three rats died while under anesthesia, leaving 51 rats (94%) which successfully recovered from surgery.

### Behavioral apparatus

All procedures took place in 32 cm × 25 cm × 21 cm conditioning chambers (Clever Systems Inc., Reston, VA) which were located within sound attenuating 45.7-cm × 43.2-cm × 43.2-cm isolation boxes (Clever Sys. Inc.). During extinction training and extinction recall sessions, the context was altered to appear different than the original conditioning context. Context A (fear conditioning) contained 28-V, incandescent, house light bulbs (Chicago Miniature Lighting, UK), whereas Context B (extinction training, extinction recall testing, and fear recall testing) contained infrared LED lights (Univivi IR Illuminator, Shenzen, China; U48R). Furthermore, while Context A had shock grid floors with stainless steel and Plexiglas walls, Context B contained painted, metal inserts placed over the floor and walls. Context B was also altered in shape by placing a bent 33.5 × 21.3-cm metal insert inside the default conditioning chambers. In addition, in Context A, chambers were wiped down with 5% acetic acid, while in Context B chambers were wiped down with 5% ammonium hydroxide. Lastly, in Context B, rats were carried into the testing room in buckets as opposed to rolled in their home cages on carts. Overhead cameras recorded behavioral sessions, and the video signal in each chamber fed into software (FreezeScan 2.00, Clever Sys. Inc., Reston, VA) that scored freezing behavior based on pixel change. Parameters have been chosen such that computer-scored freezing behavior closely matches hand-scored behavior by a trained observer. Values indicating percent duration of time spent freezing were collapsed across 30 s bins.

### Behavioral procedures

All behavioral procedures took place during the light portion of the light/dark cycle. Rats were handled for 5 days prior to the beginning of behavioral procedures and were carted into the behavioral room during the last three days of handling. On the first day of behavioral testing, an extinction recall group of rats was exposed to fear conditioning where they were placed into Context A, received a 6-min habituation period where no stimuli were presented, and then received two combinations of a 4 kHz, 76 dB, 30 s tone and a co-terminating, 1.0 mA, 1 s foot-shock (2 min ITI). In all behavioral sessions, rats were returned to their home cages 2 min following the final stimulus presentation. The following day, rats in the extinction recall group were placed into the Context B chamber and were exposed to 20 presentations of the tone (2 min ITI) as extinction training following a 6-min habituation period. The next day, rats in the extinction recall group were exposed to 4 presentations of the tone in Context B as an extinction recall test following a 6-min habituation period. 60 min following the end of the behavioral session, rats in the extinction recall group were perfused. One group of fear recall control rats was exposed to the same procedure during the first day of fear conditioning in Context A. 48 h later, these rats were placed into the Context B chamber and were exposed to 4 presentations of the tone (2 min ITI) as a fear recall test following a 6-min habituation period. 60 min following the end of the behavioral session, these rats were perfused. One group of home cage control rats remained in their home cages throughout the duration of the experiment and were perfused on the same day as the experimental rats. Each of the two cohorts of rats was split into two runs, and the number of animals in each group was balanced across runs. One rat from the fear recall group was excluded from analyses because it failed to express evidence of fear conditioning (froze less than 15% of the time during the fear recall test). See schematic representation of the behavioral timeline in Fig. 2A.

### Immunofluorescence

Rats were overdosed with Fatal Plus Solution (100 mg/kg) and perfused with ice-cold 10% PBS followed by 10% buffered formalin. The brains were extracted and stored in a solution of 30% sucrose-formalin at 4 °C for approximately 1 week. Brains were then frozen and sectioned on a cryostat, and slices were taken at 40 μm thickness. Sections were stored in a serial order in 10% PBS at 4 °C. Immunofluorescence was then performed on free-floating sections containing the brain areas of interest. Sections were washed 3 times in 10% PBS for 5 min each. Next, sections were incubated in 5% normal goat serum blocking solution for 2 h at room temperature followed by 3 additional 5-min-long washes in 10% PBS. The sections were then incubated in primary antibody (c-Fos, #2250, 1:500) (Cell Signaling, Danvers, MA) diluted in a solution of 1% BSA in 10% PBS at 4 °C over-night. The next day, the sections were washed in 10% PBS at 4 °C for 30 min, followed by 3, 5-min-long washes with 10% PBS, and incubation in secondary antibody (Alexa Fluor 594 goat anti-rabbit, red conjugate, 1:500) (Invitrogen, Carlsbad, CA) at room temperature for 2 h. After 3 more 5-min-long washes in 10% PBS, the sections were mounted onto slides and cover-slipped with Fluoromount-G (Invitrogen). See representative images of the immunostaining in Fig. 3G.

### Image acquisition

Fluorescence microscopy using an Infinity3 digital camera (Lumenera, Ottawa, ON, Canada) with a light engine (Lumencor, Beaverton, OR) attached to a Zeiss microscope was used to acquire images from each brain area of interest, including slices containing the IL which did not undergo immunofluorescence, to confirm the correct placement of the injection site. Images used for cell counting were taken at 20 × magnification. For each slice of tissue, one image was taken using a filter allowing visualization of GFP, one image was taken using a filter allowing visualization of the red conjugate of Alexa Fluor in the secondary antibody, and an imaging acquisition software (Infinity Analyze, Version 3) was used to overlay the images. All images across all brain areas were acquired with the same exposure time and gain setting. Six rats were excluded from analyses because the primary spread of the virus was outside of the IL (88% hit rate). Eight additional rats were excluded because although the virus hit the IL, they failed to show sufficient expression of GFP in all of the target brain areas of interest. Furthermore, one rat was excluded because of poor tissue quality.

### Cell counting

**Figure 1:**
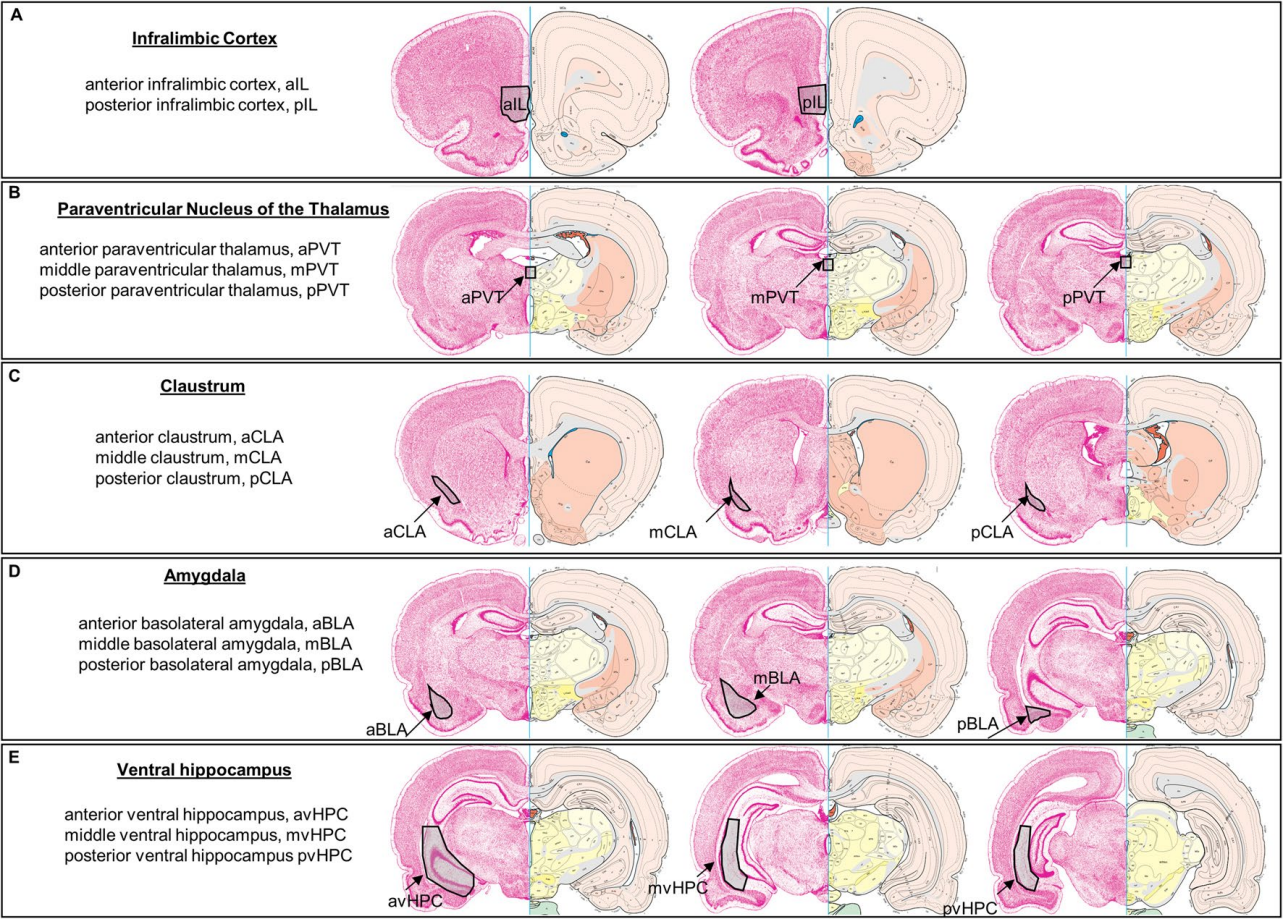
Abbreviations and locations of brain areas of interest. Explanation of abbreviations and locations for brain areas referenced throughout the manuscript. The open access brain maps are reproduced from Swanson (2004) Brain maps: structure of the rat brain, 3rd edition; under the terms of a Creative Commons Attribution-Non Commercial 4.0 International License (https://creativecommons.org/licenses/by-nc/4.0/) and are available for download at https://larrywswanson.com.

Brightness and contrast were adjusted to reduce background noise in Image J (NIH) using an identical procedure for each image. Cell counts for total number of retrogradely-labeled cells, total number of Fos-labeled, and total number of double-labeled cells were performed manually using Image J’s “Cell Counter” plugin by an experimenter who was blinded to the identity of the animals. Cell counts were normalized to cells/mm^2^. For the analysis of Fos expression in IL-projecting cells, the number of double-labeled cells was normalized to the total number of retrogradely-labeled cells. For analysis of the mBLA, mvHPC, and pvHPC, cell counts from multiple 20 × images were added together and normalized to cells/mm^2^. For analysis of the rest of the brain areas, one 20 × image or a sub-portion of one 20 × image was analyzed and normalized to cells/mm^2^. Analysis of the vHPC included the CA1, CA2, and subiculum areas of the vHPC. Figure 1 depicts the brain regions analyzed with images depicting boundaries across the anterior–posterior plane.

### Experimental design and statistical analysis

Percent time spent freezing was averaged across the 30 s periods during which the tone was playing, not including intertrial intervals. Extinction recall scores were calculated by expressing the percent time freezing during extinction recall as a percentage of freezing during the first 4 trials of extinction training (freezing during four extinction recall tones/freezing during first four extinction training tones * 100). Low scores indicated good extinction recall, whereas high scores indicated poor extinction recall. The rats’ extinction recall scores were rank-ordered, and the rats which fell within the top third of extinction recall scores were classified as “poor extinction rats”, whereas the rats which fell within the bottom two thirds of extinction recall scores were classified as “good extinction recall rats”.

Non-parametric tests were used because data often violated assumptions of normal distribution and/or homogeneity of variance. Spearman’s rank order correlations were used to determine if there was a significant association between extinction recall scores and both Fos labeling and double-labeling in the brain areas of interest among all rats which had extinction recall tests. Mann–Whitney U tests were used to determine whether there were differences between two independent groups. Kruskal–Wallis tests were used to determine whether 2 or more groups were different from one another, and Dunn’s multiple comparisons tests were used when the Kruskal–Wallis statistic was significant. A repeated-measures ANOVA with group as a between-subjects factor and trial as a within-subjects factor was used to assess freezing during extinction training. Results were considered significant when *p* < 0.05 for all statistical tests.

### Behavioral results

**Figure 2:**
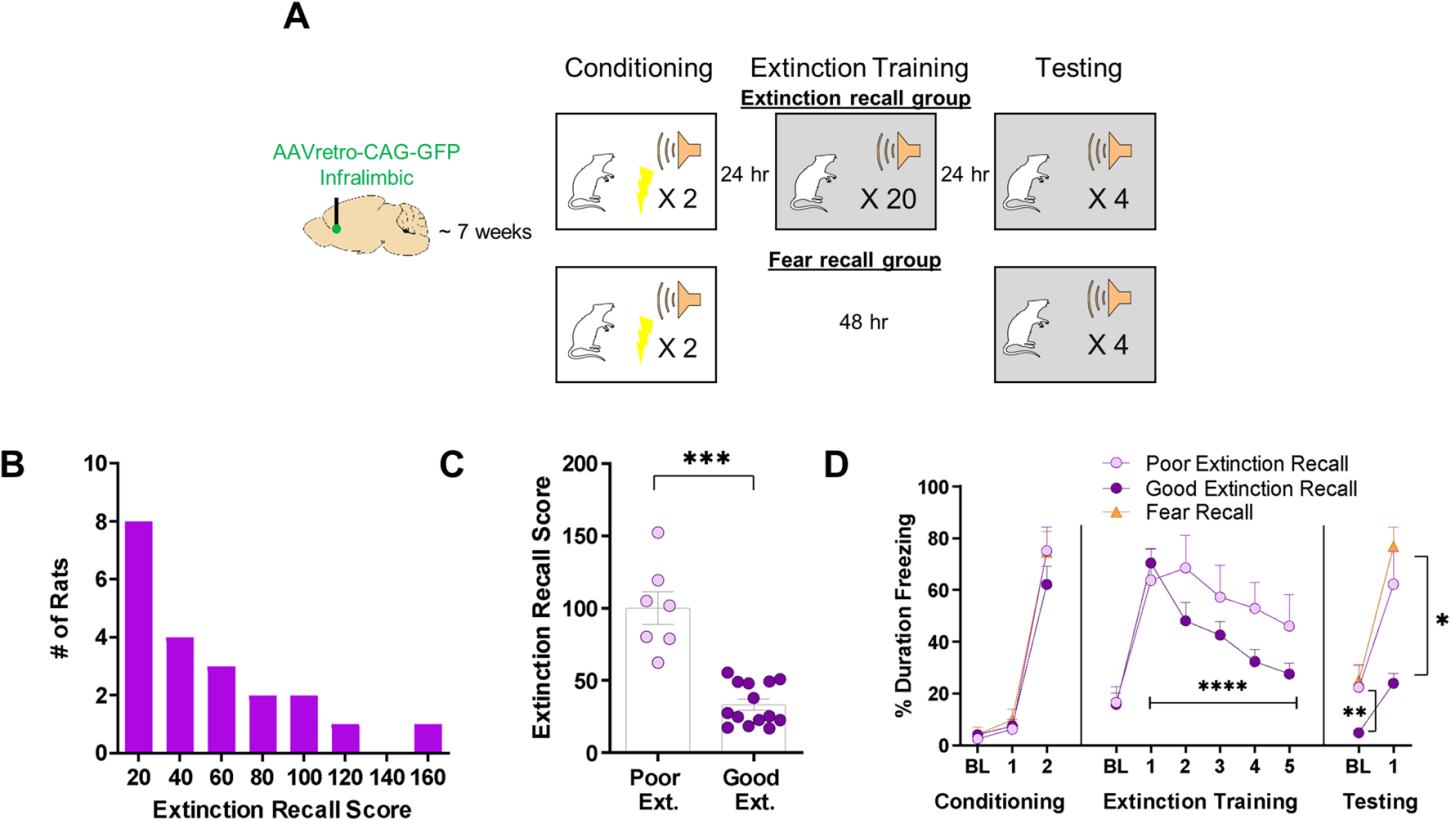
Individual variation in extinction recall. (**A**) Schematic representation of surgical and behavioral procedures. (**B**) Frequency distribution demonstrating individual variation in extinction recall scores. (**C**) Evidence that groups created on the basis of calculated extinction recall scores represent two distinct phenotypes. (**D**) Average percent time spent freezing for poor extinction, good extinction, and fear recall rats during 30 s bins in the fear conditioning session, during 20, 30-s tones collapsed into 5 blocks (4 tones each) in the extinction training session, and during four tones in the extinction recall and fear recall sessions. Error bars represent standard error of the mean. *p < 0.05, **p < 0.01, ***p < 0.001, ****p < 0.0001.

Figure 2 depicts the experimental timeline (Fig. 2A), and a frequency distribution for all rats that underwent extinction (Fig. 2B). Rats in the good and poor extinction groups differed significantly in these calculated extinction recall scores (*U* = 0, *p* < 0.001) (Fig. 2C). There was no significant difference among the good extinction, poor extinction, and fear recall groups in time spent freezing during the baseline period of the fear conditioning session (X^2^(2) = 2.746, *p* = 0.253) (Fig. 2D). Furthermore, there was neither a significant difference among the good extinction, poor extinction, and fear recall groups in time spent freezing during the first tone presentation of the fear conditioning session (X^2^(2) = 1.107, *p* = 0.575), nor was there a significant difference among the good extinction, poor extinction, and fear recall groups in time spent freezing during the second tone presentation of the fear conditioning session (X^2^ (2) = 2.214, *p* = 0.331) (Fig. 2D). There was also no significant difference between the good and poor extinction groups in time spent freezing during the baseline period of the extinction training session (*U* = 45.00, *p* = 0.799) (Fig. 2D). Next, there was a significant main effect of trial block (5 tones per block) on time spent freezing during the extinction training session (F (2.884, 54.80) = 8.331, *p* < 0.001), indicating that extinction learning occurred (Fig. 2D). However, there was no main effect of extinction group (F (1, 19) = 3.091, *p* = 0.095) on time spent freezing throughout the extinction training session, nor was there an interaction between trial block and extinction group (F (4, 76) = 1.890, *p* = 0.121) (Fig. 2D). During the testing session, there was a significant difference among the good extinction, poor extinction, and fear recall groups in time spent freezing during the baseline period (X^2^ (2) = 8.569, *p* = 0.014) such that the fear recall group froze significantly more than the good extinction group (*Mean Rank Diff.* = 10.57, *p* = 0.017), but not the poor extinction group (*Mean Rank Diff.* = − 3.714, *p* > 0.999) (Fig. 2D). The good extinction, poor extinction, and fear recall groups also differed significantly from one another in time spent freezing during the tone presentations of the testing session (X^2^ (2) = 14.93, *p* = 0.001) such that the good extinction group froze significantly less than both the poor extinction group (*Mean Rank Diff.* = 9.286, *p* = 0.044) and the fear recall group (*Mean Rank Diff.* = 13.86, *p* = 0.001) (Fig. 2D).

### Anatomical results

**Figure 3:**
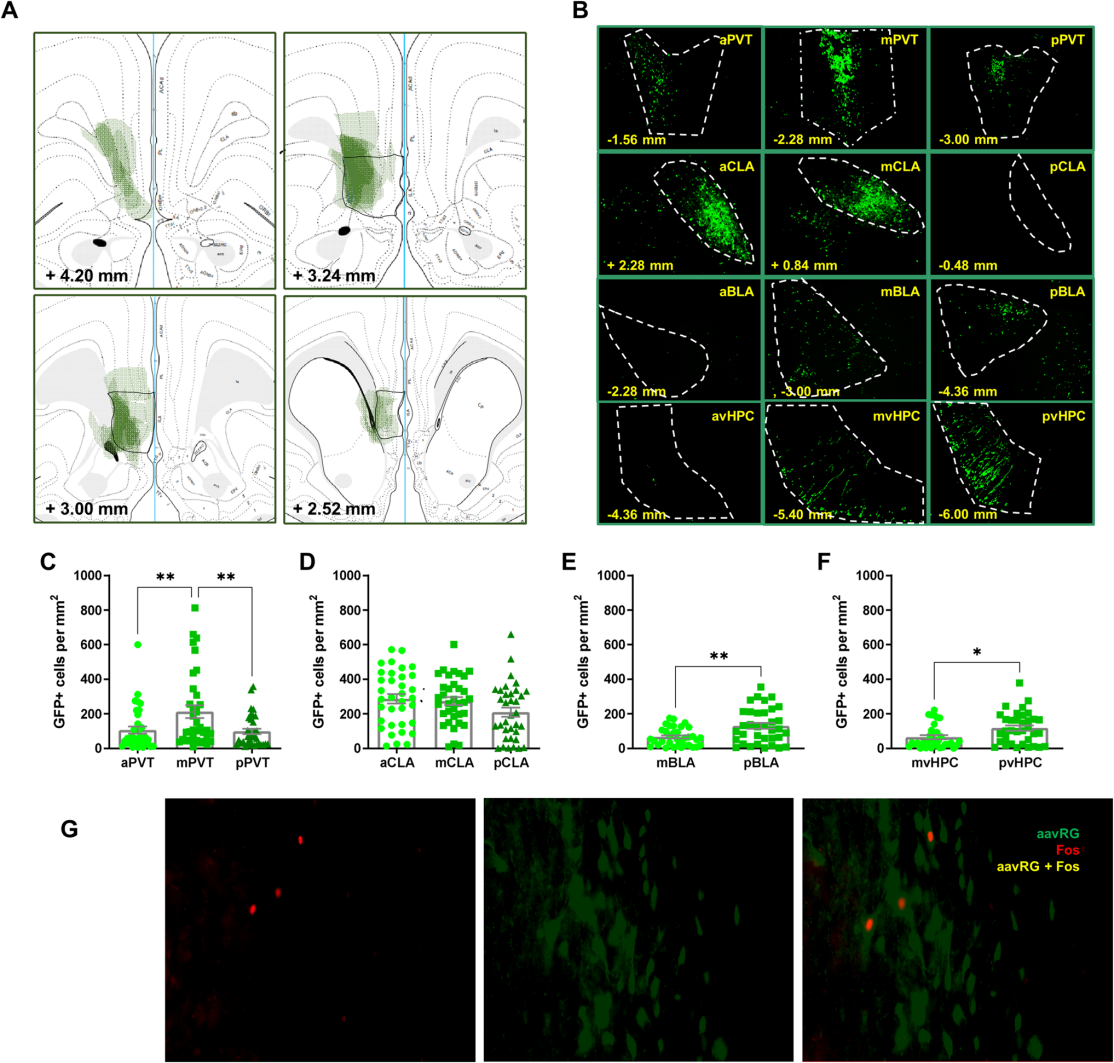
Quantification of IL-afferents throughout the brain areas of interest. (**A**) Schematic representation of the aavRG-CAG-GFP spread throughout the IL of included rats. (**B**) Representative images of retrograde labeling in different anteroposterior locations of brain areas of interest. Quantification of retrograde label along the anteroposterior axis of the (**C**) paraventricular thalamus, (**D**) claustrum, (**E**) basolateral amygdala, and (**F**) ventral hippocampus. (**G**) Representative images demonstrating aavRG retrograde labeling, Fos labeling, and double-labeling of aavRG and Fos in the aPVT. Error bars represent standard error of the mean. *p < 0.05, **p < 0.01. Scale bar 100 µm. The open access brain maps in Panel A are reproduced from Swanson (2004) Brain maps: structure of the rat brain, 3rd edition; under the terms of a Creative Commons Attribution-Non Commercial 4.0 International License (https://creativecommons.org/licenses/by-nc/4.0/) and are available for download at https://larrywswanson.com.

A retrograde tracer was infused into the IL (Fig. 3A), and quantitative analysis was performed on the number of GFP + cells along the anteroposterior axis of regions of interest (Fig. 3B–F). There was a significant difference in the number of GFP + cells among the anterior, middle, and posterior PVT (X^2^ (2) = 8.200, *p* = 0.017) such that the mPVT displayed significantly more GFP + cells than both the aPVT (*Mean Rank Diff.* = 18.37, *p* = 0.035) and the pPVT (*Mean Rank Diff.* = 17.71, *p* = 0.045) (Fig. 3C). Although multiple animals did not display any GFP + cells in the pCLA, yielding activity mapping in this area unfeasible, there was no significant difference among the anterior, middle, and posterior CLA (X^2^ (2) = 5.596, *p* = 0.061) in the number of GFP + cells (Fig. 3D). Next, because very few rats displayed any GFP + cells in the aBLA or the avHPC, only the middle and posterior portions of these regions were analyzed. There was a significant difference between the middle and posterior BLA (*U* = 393, *p* = 0.009) in the number of GFP + cells such that the pBLA displayed more IL-projections than the mBLA (Fig. 3E). Similarly, there was a significant difference between the middle and posterior vHPC such that the pvHPC displayed more IL-projections than the mvHPC (*U* = 403.5, *p* = 0.014) (Fig. 3F). Figure 3G depicts example images showing Fos, aavRG-GFP, and dual labeled cells.

### Activity mapping results

#### The paraventricular nucleus of the thalamus

**Figure 4:**
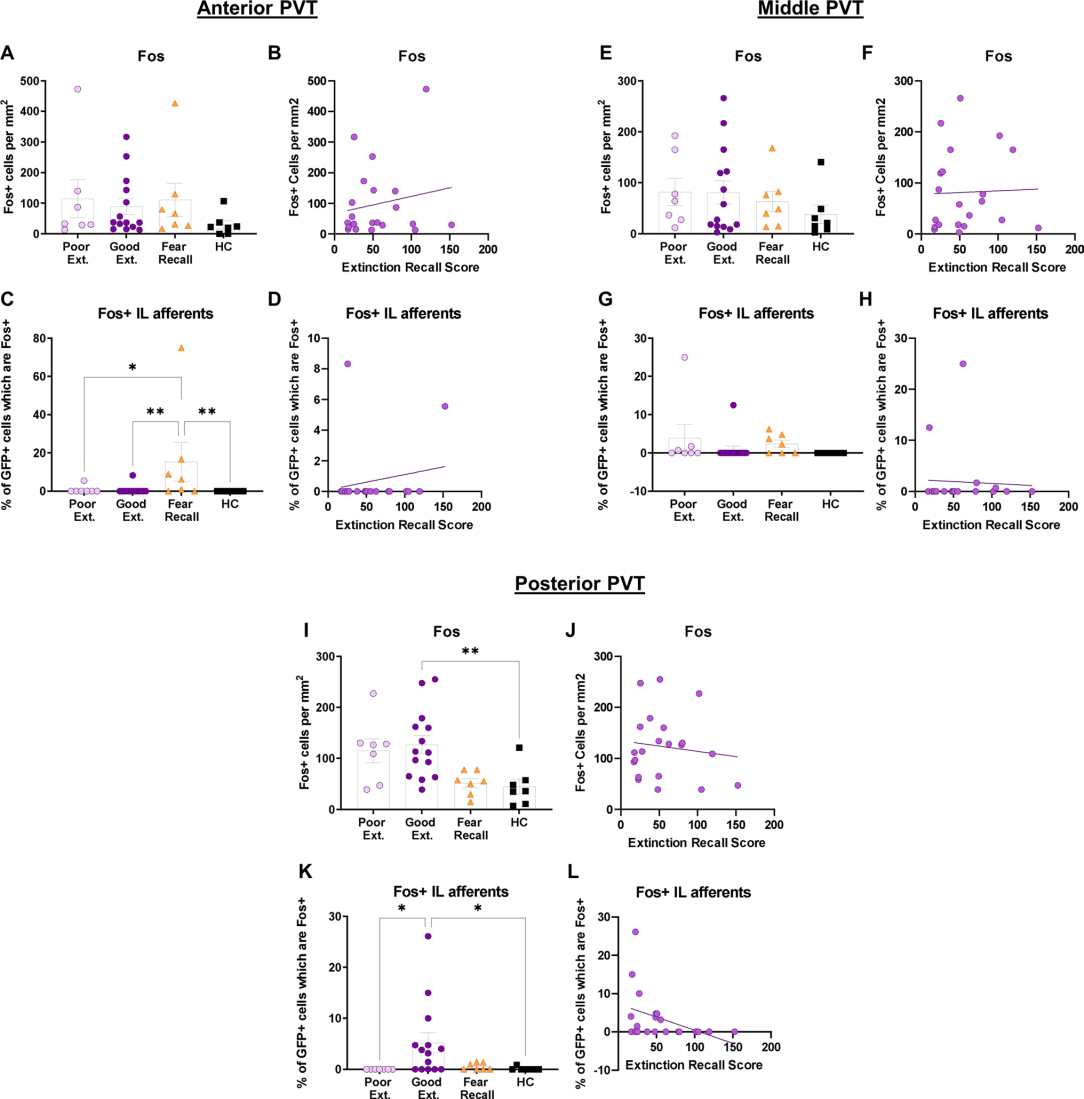
Fos activity is elevated in the IL-afferents of the posterior paraventricular thalamus (PVT) in rats displaying good extinction. (**A**) No significant differences among groups in Fos expression in the aPVT. (**B**) No significant correlation between Fos expression and extinction recall in the aPVT. (**C**) Fear recall group showed elevated Fos expression within IL-afferents relative to all other groups. (**D**) No significant correlation between Fos expression within IL-afferents and extinction recall in the aPVT. (**E**) No significant differences among groups in Fos expression in the mPVT. (**F**) No significant correlation between Fos expression and extinction recall in the mPVT. (**G**) No significant differences among groups in Fos expression within IL-afferents in the mPVT. (**H**) No significant correlation between Fos expression within IL-afferents and extinction recall in the mPVT. (**I**) Good extinction group, but no other groups, shows elevated Fos activity in the pPVT relative to the home cage group. (**J**) No significant correlation between Fos expression and extinction recall in the pPVT. (**K**) Good extinction group shows elevated Fos expression in IL-afferents relative to the poor extinction group and the home cage group. (**L**) Significant correlation between Fos expression within IL-afferents and extinction recall such that good extinction recall is related to more Fos expression within IL-afferents. Error bars represent standard error of the mean. *p < 0.05, **p < 0.01.

Both global and IL-projection specific Fos activity were analyzed in the aPVT, the mPVT, and the pPVT in all rats. There was no significant difference among the good extinction, poor extinction, fear recall, and home cage groups in Fos expression in the aPVT (X^2^ (3) = 3.888, *p* = 0.274) (Fig. 4A), nor was there a significant correlation between Fos expression in the aPVT and extinction recall (r_s_ = 0.092, *p* = 0.691) (Fig. 4B) or between Fos expression within IL-afferents of the aPVT and extinction recall (r_s_ = 0.143, *p* = 0.537) (Fig. 4D). However, there was a significant difference among good extinction, poor extinction, fear recall, and home cage groups in Fos expression within IL-afferents of the aPVT (X^2^ (3) = 15.05, *p* = 0.002) such that the fear recall group displayed more activation of IL-afferents relative to the good extinction (*Mean Rank Diff.* = 11.54, *p* = 0.003), poor extinction (*Mean Rank Diff.* = 10.57, *p* = 0.034), and home cage (*Mean Rank Diff.* = 12.79, *p* = 0.005) groups (Fig. 4C). Next, there was no significant difference among the good extinction, poor extinction, fear recall, and home cage groups in Fos expression in the mPVT (X^2^ (3) = 2.272, *p* = 0.518) (Fig. 4E), nor was there a significant correlation between Fos expression in the mPVT and extinction recall (r_s_ = 0.168 *p* = 0.468) (Fig. 4F). Although there was a significant difference among the good extinction, poor extinction, fear recall, and home cage groups in Fos expression within IL-afferents of the mPVT (X^2^ (3) = 9.252, *p* = 0.026), post hoc comparisons did not reveal significant differences between any two groups (Fig. 4G). Furthermore, there was no significant correlation between Fos expression within IL-afferents of the mPVT and extinction recall (r_s_ = 0.174, *p* = 0.450) (Fig. 4H). Next, there was a significant difference among the good extinction, poor extinction, fear recall, and home cage groups in Fos expression in the pPVT (X^2^ (3) = 13.89, *p* = 0.003), such that the good extinction group (*Mean Rank Diff.* = 14.96, *p* = 0.010), but not the poor extinction (*Mean Rank Diff.* = 12.86, *p* = 0.113) or fear recall group (*Mean Rank Diff.* = 2.571, *p* > 0.999), displayed more Fos expression than the home cage group (Fig. 4I). There was, however, no significant correlation between Fos expression in the pPVT and extinction recall (r_s_ = 0.051, *p* = 0.825) (Fig. 4J). Finally, there was both a significant difference among the good extinction, poor extinction, fear recall, and home cage groups in Fos expression within IL-afferents of the pPVT (X^2^ (3) = 12.34 *p* = 0.006) such that the good extinction group displayed more Fos expression in IL-afferents than both the poor extinction (*Mean Rank Diff.* = 12.54, *p* = 0.014) and home cage (*Mean Rank Diff.* = 12.89, *p* = 0.049) groups (Fig. 4K) and a significant correlation between activation of IL-afferents within the pPVT and extinction recall such that better extinction recall was related to more activation of these IL-afferents (r_s_ = − 0.438, *p* = 0.047) (Fig. 4L).

#### The claustrum

**Figure 5:**
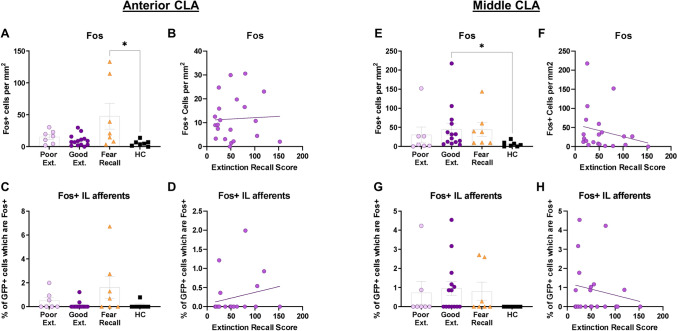
Fos activity is elevated in the middle claustrum among rats displaying good extinction recall. (**A**) Fear recall group, but not other group, shows elevated Fos activity relative to the home cage group in the aCLA. (**B**) No significant correlation between Fos expression in the aCLA and extinction recall. (**C**) No significant difference among groups in Fos expression within the IL-afferents of the aCLA. (**D**) No significant correlation between Fos expression within the IL-afferents and extinction recall in the aCLA. (**E**) Good extinction group, but not other group, shows elevated Fos activity relative to the home cage group in the mCLA. (**F**) No significant correlation between Fos expression and extinction recall in the mCLA. (**G**) No significant differences among groups in Fos expression within the IL-afferents of the mCLA. (**H**) No significant correlation between Fos expression within the IL-afferents and extinction recall in the mCLA. Error bars represent standard error of the mean. *p < 0.05.

Next, global and IL-projection specific Fos activity were analyzed in the aCLA and mCLA in rats in all groups. There was a significant difference among the good extinction, poor extinction, fear recall, and home cage groups in Fos expression in the aCLA (X^2^ (3) = 8.455, *p* = 0.036) such that the fear recall group (*Mean Rank Diff.* = 14.50, *p* = 0.049), but neither the poor (*Mean Rank Diff.* = 10.21, *p* = 0.373) nor good extinction (*Mean Rank Diff.* = 4.607, *p* > 0.999) groups, displayed more Fos expression than the home cage group (Fig. 5A). There was no significant correlation between global Fos expression in (r_s_ = 0.036, *p* = 0.876) (Fig. 5B) or Fos expression within (r_s_ = − 0.282, *p* = 0.215) the IL-afferents of the aCLA and extinction recall (Fig. 5D), nor was there a significant difference among the good extinction, poor extinction, fear recall, and home cage groups in Fos expression within the IL-afferents of the aCLA (X^2^ (3) = 6.722, *p* = 0.081) (Fig. 5C). Next, there was a significant difference among the good extinction, poor extinction, fear recall, and home cage groups in Fos expression in the mCLA (X^2^ (3) = 10.12, *p* = 0.018) such that the good extinction group (*Mean Rank Diff.* = 12.93, *p* = 0.038), but neither the poor extinction (*Mean Rank Diff.* = 5.143, *p* > 0.999) nor fear recall groups (*Mean Rank Diff.* = 14.00, *p* = 0.063) displayed significantly more Fos expression in the mCLA relative to the home cage group (Fig. 5E). However, there was no significant correlation between global Fos expression in the mCLA (r_s_ = 0.321, *p* = 0.156) (Fig. 5F) or Fos expression within the IL-afferents of the mCLA (r_s_ = − 0.121, *p* = 0.602) and extinction recall (Fig. 5H), nor was there a significant difference among the good extinction, poor extinction, fear recall, and home cage groups in Fos expression within the IL-afferents of the mCLA (X^2^ (3) = 4.923, *p* = 0.178) (Fig. 5G).

#### The basolateral amygdala

**Figure 6:**
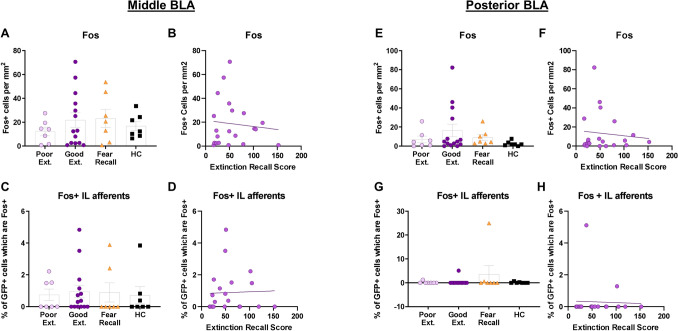
Individual variation in extinction recall does not map onto differences in Fos expression in the basolateral amygdala. (**A**) No significant differences among groups in Fos expression in the mBLA. (**B**) No significant correlation between Fos expression and extinction recall in the mBLA. (**C**) No significant differences among groups in Fos expression within the IL-afferents of the mBLA. (**D**) No significant correlation between Fos expression within the IL-afferents and extinction recall in the mBLA. (**E**) No significant differences among groups in Fos expression in the pBLA. (**F**) No significant correlation between Fos expression and extinction recall in the pBLA. (**G**) No significant differences among groups in Fos-expression within IL-afferents of the pBLA. (**H**) No significant correlation between Fos expression within IL-afferents and extinction recall in the pBLA. Error bars represent standard error of the mean.

Next, global and IL-projection specific Fos activity were analyzed in the mBLA and the pBLA in rats in all groups. There was no significant difference among the good extinction, poor extinction, fear recall, and home cage groups in Fos expression in the mBLA (X^2^ (3) = 0.944, *p* = 0.815) (Fig. 6A). There was also no significant difference among the good extinction, poor extinction, fear recall, and home cage groups in Fos expression in the IL-afferents of the mBLA (X^2^ (3) = 0.518, *p* = 0.915) (Fig. 6C). Furthermore, there was neither a significant correlation between global Fos expression in the mBLA (r_s_ = 0.126, *p* = 0.588) (Fig. 6B) nor Fos expression within the IL-afferents of the mBLA (r_s_ = 0.200, *p* = 0.385) (Fig. 6D) and extinction recall. There was also no significant difference among the good extinction, poor extinction, fear recall, and home cage groups in Fos expression in the pBLA (X^2^ (3) = 4.246, *p* = 0.236) (Fig. 6E), and no significant difference among the good extinction, poor extinction, fear recall, and home cage groups in Fos expression within the IL-afferents of the pBLA (X^2^ (3) = 1.954, *p* = 0.582) (Fig. 6G). Finally, there was neither a significant correlation between global Fos expression in the pBLA (r_s_ = 0.070, *p* = 0.762) (Fig. 6F) nor Fos expression within the IL-afferents of the pBLA (r_s_ = 0.122, *p* = 0.597) and extinction recall (Fig. 6H).

#### The ventral hippocampus

**Figure 7:**
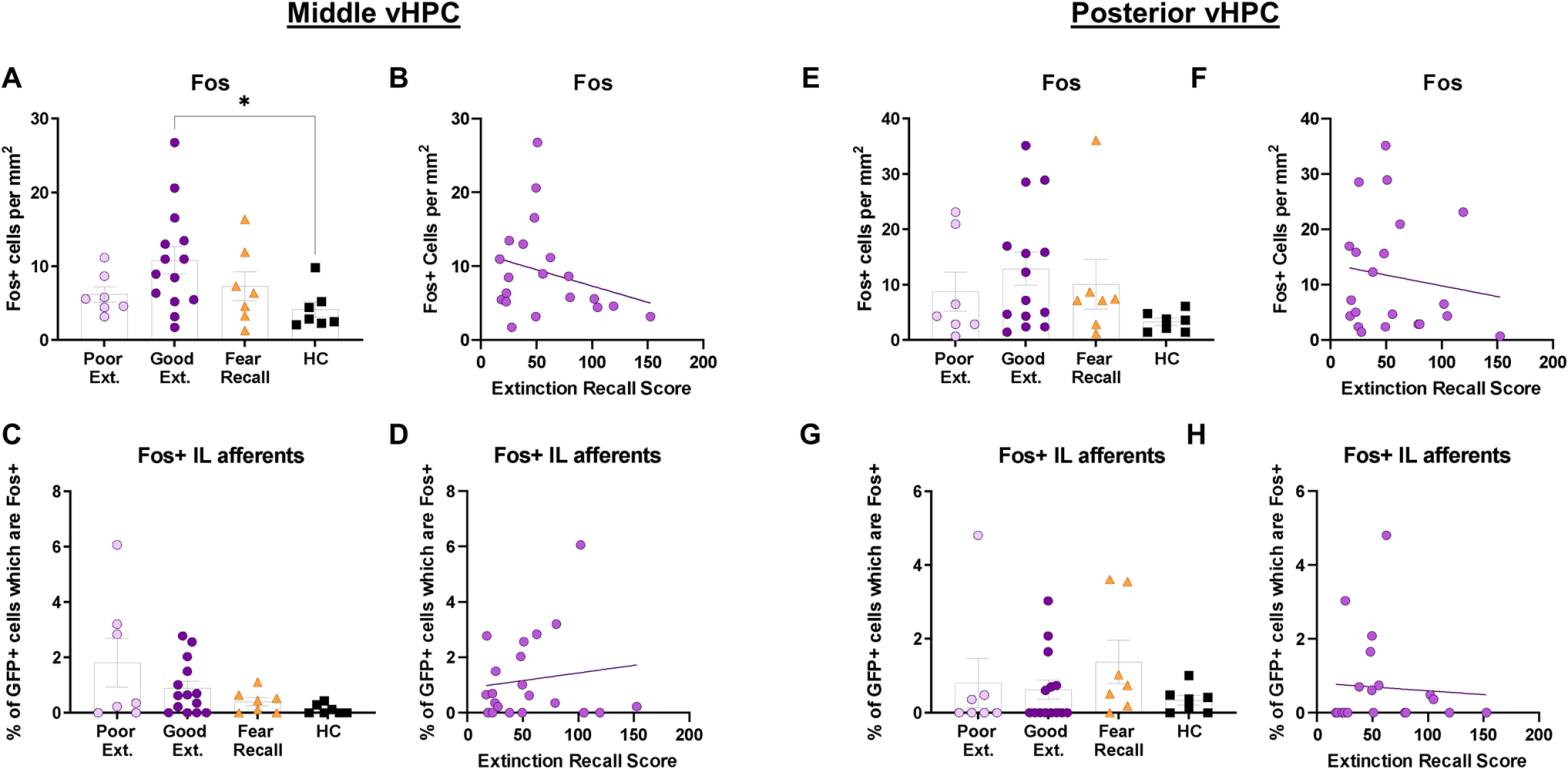
Elevated Fos expression in the middle ventral hippocampus of rats displaying good extinction recall. (**A**) Good extinction group, but not other group, shows elevated Fos expression relative to the home cage group in the mvHPC. (**B**) No significant correlation between Fos expression and extinction recall in the mvPHC. (**C**) No significant differences among groups in Fos expression within IL-afferents of the mvHPC. (**D**) No significant correlation between Fos expression within IL-afferents and extinction recall in the mvHPC. (**E**) No significant differences among groups in Fos expression in the pvHPC. (**F**) No significant correlation between Fos expression and extinction recall in the pvHPC. (**G**) No significant differences among groups in Fos expression within the IL-afferents of the pVHPC. (**H**) No significant correlation between Fos expression within IL-afferents and extinction recall in the pvHPC. Error bars represent standard error of the mean. *p < 0.05. Our primary analysis for all regions report comparisons at three levels along the anterior-posterior axis, although we also analyzed each regions collapsed across the AP axis. Results from these analyses are depicted in Table 1.

Finally, global and IL-projection specific Fos activity were analyzed in the mvHPC and the pvHPC in all rats. There was a significant difference among the good extinction, poor extinction, fear recall, and home cage groups in Fos expression in the mvHPC (X^2^ (3) = 8.056, *p* = 0.045) such that the good extinction (*Mean Rank Diff.* = 13.29, *p* = 0.031), but neither poor extinction (*Mean Rank Diff.* = 6.857, *p* > 0.999) nor fear recall (*Mean Rank Diff.* = 8.000, *p* = 0.864) groups showed more Fos expression than the home cage group (Fig. 7A). There was, however, no significant difference among the good extinction, poor extinction, fear recall, and home cage (*Mdn* = 0.000, *Range* = 0.433) groups in Fos expression in the IL-afferents of the mvHPC (X^2^ (3) = 4.893, *p* = 0.180) (Fig. 7C). Furthermore, there was neither a significant correlation between global Fos expression in the mvHPC (r_s_ = − 0.233, *p* = 0.309) (Fig. 7B) nor Fos expression within the IL-afferents of the mvHPC (r_s_ = 0.056, *p* = 0.810) (Fig. 7D) and extinction recall. Next, there was no significant difference among the good extinction, poor extinction, fear recall, and home cage groups in Fos expression in the pvHPC (X^2^ (3) = 3.623, *p* = 0.353) (Fig. 7E), and no significant difference among the good extinction, poor extinction, fear recall, and home cage groups in Fos expression within the IL-afferents of the pvHPC (X^2^ (3) = 3.871, *p* = 0.276) (Fig. 7G). Finally, there was neither a significant correlation between global Fos expression in the pvHPC (r_s_ = − 0.127, *p* = 0.584) (Fig. 7F) nor Fos expression within the IL-afferents of the pvHPC (r_s_ = 0.176, *p* = 0.447) and extinction recall (Fig. 7H).

**Table 1:** Summary of whole area comparisons in target regions.

Brain area	Kruskal–Wallis (df3)	*p* value	Dunn’s test
Whole PVT—Fos	8.84	0.032	Good extinction > HC control
Whole PVT—double-label	24.97	< 0.0001	Good extinction > HC control and fear recall Poor extinction > HC control and fear recall
Whole CLA—Fos	20.99	0.0001	Good extinction and poor extinction > fear recall
Whole CLA—double-label	4.38	0.22	Not significant
Whole BLA—Fos	2.71	0.44	Not significant
Whole BLA—double-label	0.26	0.97	Not significant
Whole vHPC—Fos	1.78	0.41	Not significant
Whole vHPC—double-label	0.57	0.75	Not significant

## Discussion

Here, we tested whether individual differences in extinction recall would be reflected in different patterns of activity in afferents to the infralimbic cortex. To this end, we assessed Fos activity in projections to the IL from the paraventricular thalamus, claustrum, basolateral amygdala, and ventral hippocampus following extinction recall. Within cells that project to the IL, we found that activity in the posterior region of the PVT was higher in rats which showed good extinction recall compared to rats with poor extinction. No differences were observed in IL afferents from the claustrum, ventral hippocampus, or basolateral amygdala. Outside of the IL-projecting cells, increased neural activity was observed in good extinction rats in select regions of the claustrum and ventral hippocampus. Our results suggest that successful extinction recall is orchestrated by specific PVT projections to the IL and non-IL targeting cells in the claustrum and ventral hippocampus.

Our finding that PVT projections to the IL are active in rats that showed good extinction recall is consistent with a recent study showing that the PVT is necessary for extinction recall^27^. This study did not employ manipulations that were sub-region specific, however they did show that both PVT projections to the lateral division of the central amygdala, and IL projections to the PVT, are necessary for extinction recall. Our results suggest that in addition to the IL-PVT-CeL circuit, posterior PVT inputs to the IL might also be required for extinction recall. Thus, it appears as though both efferent and afferent connections of the IL are involved in extinction recall. An important next step will be to determine what drives the pPVT to signal extinction recall at the neural circuit level. In addition to reciprocal connections to the IL, prior tract tracing studies^31,32^ have showed that the pPVT receives input from the ventral periaqueductal gray (vPAG), which has been implicated in extinction learning^33–36^. While the role of the vPAG in extinction recall has not been established, projections to the pPVT from the vPAG are an attractive candidate given their density and prior evidence of both regions’ involvement in the acquisition of fear extinction.

Another important aspect of our results from the PVT is that they are specialized along its anteroposterior axis. Strikingly, neural activity within PVT projections to IL were associated with opposing behavioral states such that activity in anterior PVT projections to the IL were associated with fear recall, whereas pPVT projections were active following successful recall of extinction (i.e. low fear). Such heterogeneity of function within the PVT is not surprising given prior work [discussed in 37]. A prime example of the distribution of function within the PVT came recently^38^ in a study which characterized the properties of specific cell types in the PVT. This study showed that DRD2-expressing dopamine cells, preferentially expressed in the pPVT, innervated the prelimbic cortex and were responsive to aversive stimuli. A second population of cells were predominantly expressed in the aPVT and signaled a transition into a state of low physiological arousal, and which innervated the infralimbic cortex. Our results do not easily fit this framework, as aPVT cells projecting to the IL were active during fear recall and pPVT projections were active while animals were expressing low levels of fear. There are at least two possible explanations as to the apparent discrepancy. First, the identified cell types are not exclusively located in one anteroposterior location in the PVT. Thus, it is possible that the IL-projecting pPVT cells which are active in rats displaying good extinction recall belong to the class of cells which is more likely to be found in aPVT and signal a transition to a low arousal state. The same might be true for the IL-projecting cells in the aPVT which are activated following fear recall. Second, prior tracing studies have established the presence of pPVT projections to the IL^3^, and while few appear to originate from DRD2-containing cells, it is possible that other cell types project to the IL and which are activated by successful extinction recall.

Although the goal of the present study was to identify differences among rats displaying different extinction phenotypes, these experiments also revealed novel findings pertaining to the mechanisms underlying fear recall. Of interest, we showed elevated Fos activity in the anterior portion of the CLA in rats expressing a fear memory.

The claustrum is positioned as a hub for cortical connectivity and has been implicated in processes ranging from sensory integration, to attention and sleep^40–43^. There is limited data as to how the claustrum might participate in fear conditioning or the expression of fear, however, an earlier study showed that the expression of contextual fear engages Fos activity in the claustrum^44^. More recently, it was reported that inhibiting claustral projections to the entorhinal cortex during contextual fear conditioning attenuated long-term memory formation^29^, although their requirement for the expression of fear was not tested. In the same study, increased Fos activation was observed when animals were exposed to a novel context compared to mice exposed to a familiar context. Given this, it is possible that the activation of CLA we report here is driven by the exposure to the novel chamber during testing and not fear recall per se. To more precisely understand the function of the claustrum in fear and contextual processing, future studies should employ targeted manipulations of claustrum.

Despite prior work implicating the PVT in fear memory expression^45–47^, we did not observe any change in overall Fos expression in rats recalling fear 48 h after conditioning. Several factors may explain the discrepancy, including the fact prior work tested fear to the discrete cue in the same context in which conditioning occurred, whereas in our experiments testing took place in a novel chamber. Additionally, we sacrificed our animals 60 min following testing, while prior work used a 90-min time point. Finally, in the prior studies testing took place in a chamber in which the animals were able to make appetitive responses, whereas in our work no appetitive responses were made while the rats were tested. While this allows for a measure of conditioned suppression, there is evidence that allowing animals the ability to bar press for food while simultaneously testing them for fear to the cue produces a motivational conflict (i.e. fear vs reward) and that this is the key factor driving the engagement of PVT^48,49^.

The basolateral amygdala is known to be involved in the acquisition of fear extinction^50,51^, and there is evidence that BLA projections to the IL are also involved in this process^23^. However, whether the BLA and its connections participate in the recall of extinction is less clear. Imaging studies^23,28^ have reported increased activity in BLA in animals recalling extinction memory. However, our prior work showed no difference in activation of the BLA in good versus poor extinction rats^9^, and our results here show that extinction recall does not drive activation in the BLA broadly or within BLA projections to the IL. Consistent with our findings, whereas circuit manipulation studies indicate that IL inputs to the BLA are important for extinction learning^21,52^, they are not necessary for extinction recall^52^. However, a role for the BLA cannot be completely dismissed as recent evidence indicates that certain cell types in the BLA are required for extinction recall^53^.

It is noteworthy that fear recall did not drive Fos activation in the BLA, as prior lesion, drug, and imaging studies have implicated this area in fear expression and/or the reconsolidation of fear after retrieval^54–57^. The data presented here combine across the basal and lateral subnuclei of the amygdala and prior data has shown that fear expression drives Fos activity specifically in the dorsal part of the lateral nucleus^28^. We did analyze the basal and lateral data separately, however there were no differences in either case (data not shown) and we collapsed across both areas in the data presented here. We did not analyze subregions within the lateral amygdala, and therefore it is possible that specific changes within this area were obscured. Another possibility for the lack of a change in Fos activity in the BLA relates to the timing of fear recall relative to conditioning. Some prior work has shown that the contribution of the BLA to fear expression decreases as a function of time after conditioning such that expression depends on BLA 24 h after conditioning, but is independent of the BLA by 7 days (Ref.^45^, but see^58^). Our recall test occurred 48 h after learning, making it possible that the lack of change in Fos activity at this time point reflects a time-dependent change in the participation of the BLA in fear expression.

Finally, we found evidence that successful extinction recall involves the ventral hippocampus. This was specific to the ‘middle’ vHPC, as the same pattern was not observed in posterior regions. Consistent with prior work^59^, we did not detect changes in Fos activation in vHPC afferents to the IL. There is considerable evidence that the renewal of fear seen when the CS is encountered outside the context in which extinction occurs requires the vHPC^28,60,61^ and that it depends at least partially on vHPC inputs to the IL^13^. Based on these prior findings, we might have expected that poor extinction would be associated with increased activity in vHPC projections to the IL. However, this was not the case as there was no difference in Fos activity either in retrograde-labeled vHPC projections to IL or in non-labeled cells in the vHPC. This suggests that the failure to recall extinction in the extinction context may recruit different mechanisms than the renewal of fear.

It is important to note some limitations inherent in the design and analyses and how they might impact our conclusions. First, we divided the rats into ‘good’ and ‘poor’ by grouping animals into the top two-third vs bottom-third based on extinction recall scores. This was done to avoid grouping arrangements that would either split animals from the middle of the distribution into separate groups or would exclude animals from the middle of the distribution, such as would be the case with a median split or comparing top and bottom third of rats. We wished to avoid this because a median split would not capture the variability in response to trauma in humans, which is what we are attempting to model. Moreover, while comparing the top and bottom third of rats would allow us to compare groups of equal sizes, this approach ignores animals in the center of the distribution and also does not accurately reflect variability in response to trauma. While our approach may present issues with heterogeneity of variance and comparing groups with unequal sample sizes, it captures what we are trying to emulate better than alternative approaches.

The findings reported here help us to better understand how individual differences in extinction recall are reflected in differences in neural circuit activity. Our results might be relevant to post-traumatic stress disorder, which is known to involve excessive fear and a failure to extinguish fear responses. We showed that differences in extinction recall were associated with differences in neural activity both within, and outside, of projections to the IL. These differences were organized in discrete regions along the anteroposterior axis, further highlighting the importance of assessing brain function at the sub-region level. Shortcomings of the current approach include the correlational nature of the study and a focus on male rodents. Future studies should determine the neurobiological mechanisms of extinction learning in female rodents and employ approaches which allow for causal inferences.

## Data Availability

The datasets used and/or analyzed during the current study available from the corresponding author on reasonable request.
